# Upregulation of C/EBP Homologous Protein induced by ER Stress Mediates Epithelial to Myofibroblast Transformation in ADTKD-UMOD

**DOI:** 10.7150/ijms.65036

**Published:** 2022-01-24

**Authors:** Dan Wang, Yagui Qiu, Jinjin Fan, Yuanying Liu, Wenfang Chen, Zhijian Li, Wei Chen, Xin Wang

**Affiliations:** 1 Department of Nephrology, The First Affiliated Hospital, Sun Yat-sen University, China.; 2 Key Laboratory of Nephrology, Ministry of Health and Guangdong Province, China.; 3 Department of Pathology, The First Affiliated Hospital, Sun Yat-sen University, China.

**Keywords:** CHOP, ER stress, EMT, ADTKD-UMOD, Renal fibrosis

## Abstract

Autosomal dominant tubulointerstitial kidney disease due to *UMOD* mutations (ADTKD-UMOD) results in chronic interstitial nephritis, which gradually develops into end-stage renal disease. It is believed that the accumulation of mutant uromodulin causes the endoplasmic reticulum (ER) stress, then leads to the kidney damage. But the underlying mechanism remains unclear. To find the ADTKD-UMOD patients, *UMOD* gene screening was performed in 26 patients with unexplained chronic interstitial nephritis, during the past 10 years in our department, and among them three ADTKD-UMOD cases were discovered. Routine pathological staining and electron microscopy sections were reviewed again to confirm their kidney lesions. Immunostaining of UMOD and ER stress marker GRP78, as well as CHOP have all been done. The strong colocalization of UMOD with GRP78 and CHOP in ADTKD-UMOD patients but not in other chronic interstitial nephritis patients had been found. Moreover *in vitro* experiments, ER stress induced by tunicamycin (TM) not only significantly increased the expression of GRP78 and CHOP, but also caused the epithelial to myofibroblast transformation (EMT) of renal tubular epithelial cells, evidenced by decreased expression of E-cadherin and increased expression of vimentin, and extracellular matrix (ECM) deposition, evidenced by increased expression of fibronectin (FN). *CHOP* knockdown could restore the upregulation of vimentin and FN induced by TM. Thus, specific activation of CHOP in renal tubular epithelial cells induced by UMOD protein might be the key reason of renal interstitial fibrosis in ADTKD-UMOD patients.

## Introduction

ADTKD-UMOD is an autosomal dominant tubulointerstitial kidney disease caused by *UMOD* pathogenic mutations 1. The main manifestations are unexplained chronic kidney failure and early-onset hyperuricemia in adolescence. Because of its low diagnosis rate, there are few studies on its mechanism. Currently, it has already verified that mutant UMOD could cause ER stress both *in vivo* and *in vitro* 2, 3. The underlying mechanism is that gene mutations cause abnormal uromodulin production, which cannot undergo normal protein folding, leading to its accumulation in the ER and subsequently continuous ER stress 2, 4. In addition to ER stress, it is believed that mutant protein induces impaired ion channel function, which in turn leads to mild natriuresis and reabsorption of uric acid in the proximal renal tubules, which is the cause of secondary hyperuricemia 5.

Although it has long been demonstrated that ER stress is related to the occurrence and development of fibrotic diseases including renal fibrosis 6-10, pulmonary fibrosis 11, and cardiac remodeling 12. Until recently, C/EBP Homologous Protein (CHOP), an ER stress-related transcription factor, has been shown to be related to renal fibrosis through apoptosis and inflammatory responses 8, 13. However, whether CHOP also plays a role in the renal fibrosis of ADTKD-UMOD is still unclear. Our study will explore the underlying mechanism of CHOP in ADTKD-UMOD both *in vivo* and *in vitro*.

## Materials and methods

### Participants and clinical data

Screening criteria for the study 14: 1. Patients who underwent renal puncture examination in the First Affiliated Hospital, Sun Yat-sen University between November 2011 to September 2019, and the renal pathological diagnosis was chronic interstitial nephritis or sclerosing glomerulonephritis, with negative immune deposition; 2. History of hyperuricemia or gout; 3. Urinalysis: 24-h urine protein quantification < 1 g, or urine protein qualitative (negative or weakly positive), and urine red blood cells are negative. Case information including demographic, clinical and pathological data were acquired by enquiry of clinical records and telephone interviews. Renal function was calculated by The Modification of Diet in Renal Disease Study (MDRD) equation based on serum creatinine. Fractional excretion of uric acid was calculated based on the equation: (urinary uric acid c. * serum creatinine c.) /(serum uric acid c. * urine creatinine c.) (c. means concentration) 1. DNA was extracted from blood samples collected from 26 cases and all available family members of the probands. Histopathology staining images including Hematoxylin-Eosin (HE) staining and Masson's trichrome staining as well as electron microscopy images at the time of diagnosis were obtained from the pathology department. This research was approved by the ethics committee of the First Affiliated Hospital, Sun Yat-sen University. Written consents received from all participants.

### Immunofluorescence staining

Paraffin-embedded sections (5μm thick) from participants' renal biopsy tissues and a donor kidney specimen were obtained for immunofluorescence staining according to standard protocols. In brief, sections were deparaffinized, and subsequently hydrated in an alcohol gradient (100%, 95%, 75%) and distilled water. The rehydrated slides were placed in a box filled with 0.01 mol/L citrate buffer for antigen retrieval by heating for 15 min in an antigen retrieval tank (Aptum Antigen Retriever, EM Science, USA). After overnight incubation in citrate antigen retrieval solution, the slides were blocked with 2% BSA at room temperature for 1 h. Different primary antibodies were incubated overnight at 4°C, and after washing, the corresponding secondary antibodies were incubated at room temperature for 1 h. After a few washes, the slides were stained with DAPI (Thermo Fisher, USA, 1: 500, D21490) for 2 min, and finally mounted with anti-quench glass solution (ProLong™ Glass Antifade Mountant, P36980). A laser confocal fluorescence microscope (Zeiss800, Carl Zeiss, Germany) was used to detect positive signals. Identical parameters were used for different kidney sections when acquiring images. The primary antibodies used were: polyclonal sheep anti-human UMOD antibody (R&D Systems, USA; Cat #AF6144, 1:100), proximal tubule brush border labeling antibody LTL-488 15(Fluorescein Lotus Lectin, Vector Laboratories, USA, FL-1321; 1:1000), anti-GRP78 BiP antibody (abcam, Cambridge, UK; ab21685; 1:500) and CHOP (L63F7) mouse mAb (Cell Signaling Technology, Danvers, MA, USA; #2895; 1:50). Secondary antibodies included Alexa Fluor 546 donkey anti-sheep IgG antibody, Alexa Fluor 488 donkey anti-mouse IgG antibody, Alexa Fluor 488 donkey anti-Rabbit IgG antibody and Alexa Fluor 647 donkey anti-Rabbit IgG antibody (Invitrogen, Carlsbad, CA, USA).

### DNA extraction and Sanger sequencing

DNA samples isolated from whole blood of participants were obtained from the Biological Sample Bank of First Affiliated Hospital, Sun Yat-sen University. DNA samples were abstracted from whole blood of their available family members by TsingKe Biological Technology company (Beijing, China). One hundred nanograms of genomic DNA was used for PCR amplification of the *UMOD* exons area, using primers designed with Primer-BLAST (NCBI). Sanger sequencing was conducted at TsingKe Biological Technology company according to the standard method.

### Cell culture and treatment

For *in vitro* experiments, human renal tubular epithelial cells (HK2) and mouse renal tubular epithelial cells (mTEC) (acquired from ATCC, American Tissue Culture Collection) were cultured in DMEM/F12 containing 10% FBS, 100 U/mL penicillin and 100 μg/mL streptomycin at 37 °C in a 5% CO_2_/95% air atmosphere. The cells were treated with tunicamycin (abcam, ab120296) or control DMSO and ER stress, EMT markers and ECM protein were examined. Additionally, HK2 and mTEC were transfected with two different small interference RNA (siRNA) against *CHOP* or control siRNA for 24 h, and then treated with tunicamycin for another 24 h (for mRNA detection) or 48 h (for protein detection ) to explore the effect of CHOP on EMT and ECM protein of tubular cells under ER stress.

### Transfection of siRNA

Transfections were conducted with Lipofectamine 3000 (Invitrogen) for HK2 and HiPerFect Transfection Reagent (QIAGEN) for mTEC according to the manufacturers' protocols, with a final concentration of siRNA of 100 nM. *CHOP* siRNA and negative control siRNA were synthesized by Genomeditech company (Shanghai, China). The sequence (5′-3′) of *CHOP* siRNA: Human: siCHOP1: GUCCUGUCUUCAGAUGAAA tt and UUUCAUCUGAAGACAGGAC tt; siCHOP2: UGAACGGCUCAAGCAGGAAAU tt and AUUUCCUGCUUGAGCCGUUCA tt. Mouse: siCHOP1: GGUCCUGUCCUCAGAUGAAAU tt and AUUUCAUCUGAGGACAGGACC tt; siCHOP2: GAUUCCAGUCAGAGUUCUAUG tt and CAUAGAACUCUGACUGGAAUC tt.

### Quantitative Polymerase Chain Reaction

Total RNA was extracted from cells using a RNA Quick Purification Kit (ES Science, RN001) and cDNA synthesis was conducted using Transcriptor First Strand cDNA Synthesis Kit (Roche, No. 04897 030001). Quantitative polymerase chain reaction was performed with FastStart Universal SYBR Green Master (Roche, No. 04913914001) in LightCycle480 II (Roche). QPCR Primers sequences were synthesized by TsingKe Biological Technology company and available in Supplement File 2.

### Immunoblotting

Western blot analysis was conducted according to a standard method. In brief, cell protein was extracted with Radio Immunoprecipitation Assay Lysis Buffer (RIPA buffer, Thermo Fisher Scientific, Waltham, MA, USA) containing a protease inhibitor cocktail (Roche, 05892970001) on ice for 30 min. After homogenization and centrifugation, the protein concentration in the supernatant was quantified by a BCA assay kit, and equal amounts of protein were loaded on an SDS polyacrylamide gel, and then transferred to a polyvinylidene fluoride membrane. The membrane was blocked with 5% nonfat milk for 1 h at room temperature, and then incubated with primary antibodies at 4 °C overnight. Then, the membrane was incubated with the corresponding horseradish peroxidase (HRP)-conjugated secondary antibodies for 1 h at room temperature, followed by three washes. Immobilon western chemiluminescent HRP substrate (Millipore, Massachusetts, American) was used to detect the protein signal. Image j software was used to measure the band intensity for semi-quantitative analysis. The antibodies used in the western blots: anti-CHOP (Cell Signaling Technology, #2895), anti-GRP78 BiP antibody (abcam 21685), anti-E-cadherin (abcam 40772 for HK2, Cell Signaling Technology, #14472 for mTEC), anti-vimentin (Cell Signaling Technology, #5741) and anti-fibronectin (abcam 2413).

### Statistics analysis

Software Prism 8 version 8.0.0. was used to analyze the data and create quantitative graphs of the western blotting results and qPCR results. Student's *t* test was used for comparison between two groups in the *in vitro* experiments. Data are shown as mean ± standard error of the mean (SEM). Significant difference was defined as *P* < 0.05.

## Results

### ADTKD-UMOD was diagnosed in three patients through detection of *UMOD* gene mutations

The data of patients who underwent renal biopsy in our hospital from November 2011 to September 2019 were collected. Out of 7679 cases, 113 cases were diagnosed with chronic interstitial nephritis or sclerosing nephritis. Among them, 26 cases met the enrollment criteria performed *UMOD* exome sequencing. Three *UMOD* missense variants in three patients were found (Table 1), all of which were predicted to be harmful by Protein Variation Effect Analyzer (PROVEAN), a bioinformatics protein function prediction software 16. Two of the mutation (c.944G>C, c.377G>A) were newly identified, and one (c.1815A>G) had been reported previously 17. Both of novel mutations, c.944G>C, and c.377G>A, located in exon 3, cause the substitution of conserved cysteine residues (C315S and C126Y respectively), which break disulphide bonds (between position 300 and 315, position 112 and 126,) that were in the wild-type in EGF-like 3 domain predicted by Missense3D 18 and PROSITE 19. The structure diagram of the mutant protein (C315S) are shown in the supplementary file 3. Previously, a mutation at 315 (C315R) has been detected to cause a delay in protein export to the plasma membrane due to a longer retention time in the ER 20. Further *UMOD* genetic testing of their family members revealed the same pathogenic mutations, which is consistent with autosomal dominant inheritance.

### The clinical characteristics of ADTKD-UMOD cases

Specific clinical features of the three cases are shown in Table 2. All three patients had youth-onset hyperuricemia, and subsequently increased serum creatinine, as well as a family history of hyperuricemia. One patient (22 years old) has no obvious family history of kidney disease, but his mother also has hyperuricemia. The other two patients have a family history of chronic kidney disease (CKD). There were no prominent abnormalities in the urine test. The kidneys are normal in size. Glomerular sclerosis and interstitial fibrosis had been seen in the renal biopsies and immune deposition is all negative or mild non-specific changes, without obvious urate crystals. Multifocal fibrosis was found around the distal tubules without apparent infiltration of lymphocytes. More detailed information of the three cases including their family histories are described in Supplementary File 1.

### Mutant uromodulin accumulates in ADTKD-UMOD

**Figure 1:**
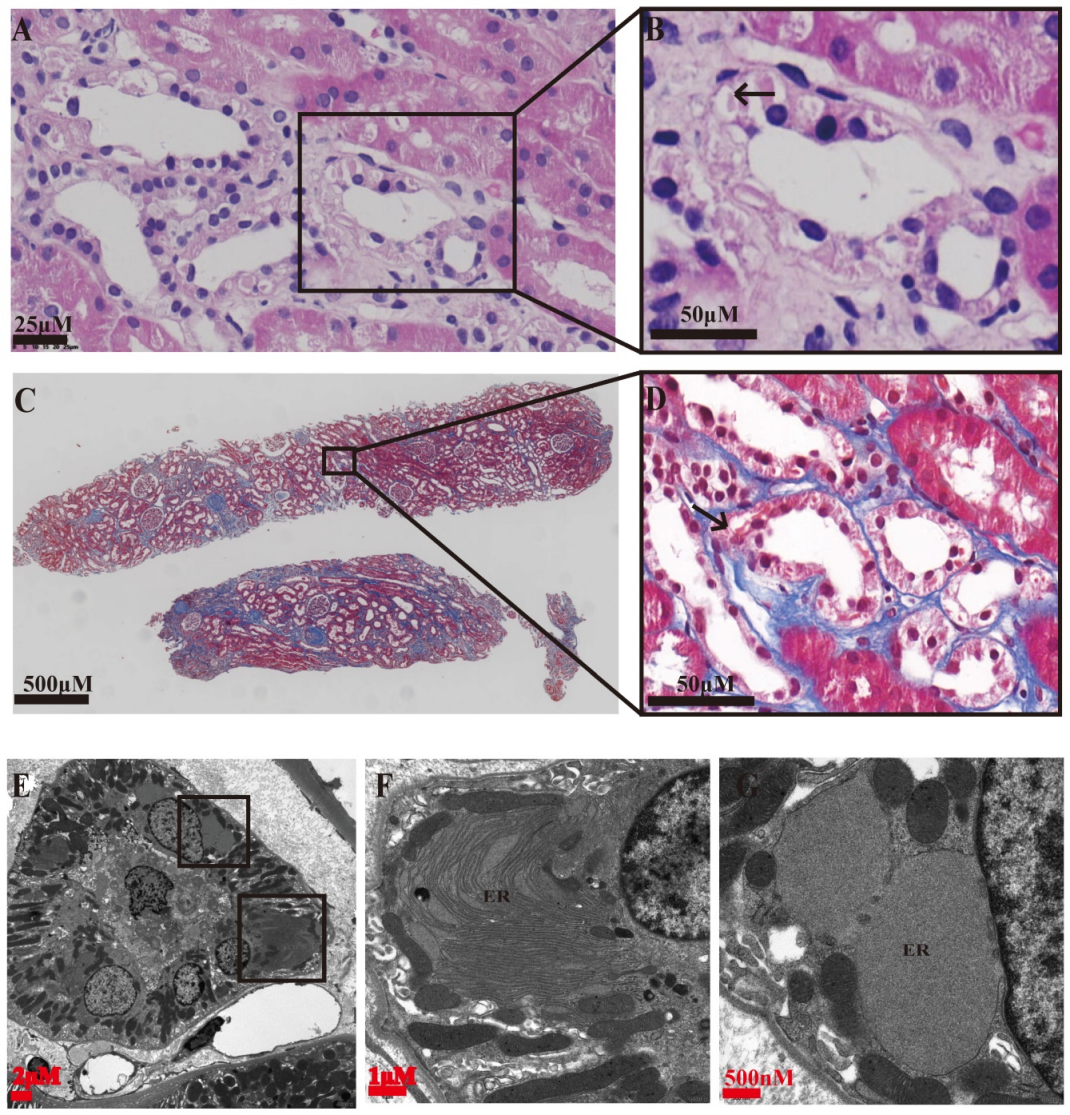
** Representative histopathology findings of an ADTKD-UMOD renal biopsy (from CASE 2). (A, B)** Hematoxylin-Eosin staining showing eosinophilic “fluffy” inclusions in thick ascending limb of Henle's loop (TALH) (arrow), i.e., abnormal protein accumulation.** (C, D)** Masson's trichrome staining showing interstitial fibrosis changes, obvious fibrosis around the distal tubules and an intracellular hyaline change (arrow). **(E-G)** Electron microscopy images; **(E)** a complete image of a distal tubular epithelium. **(F, G)** partial enlargement of **(E)**, showing that the rough ER and the smooth ER are obviously expanded, and accumulation of lower electron density in the ER, i.e., abnormal accumulation of protein.

**Figure 2:**
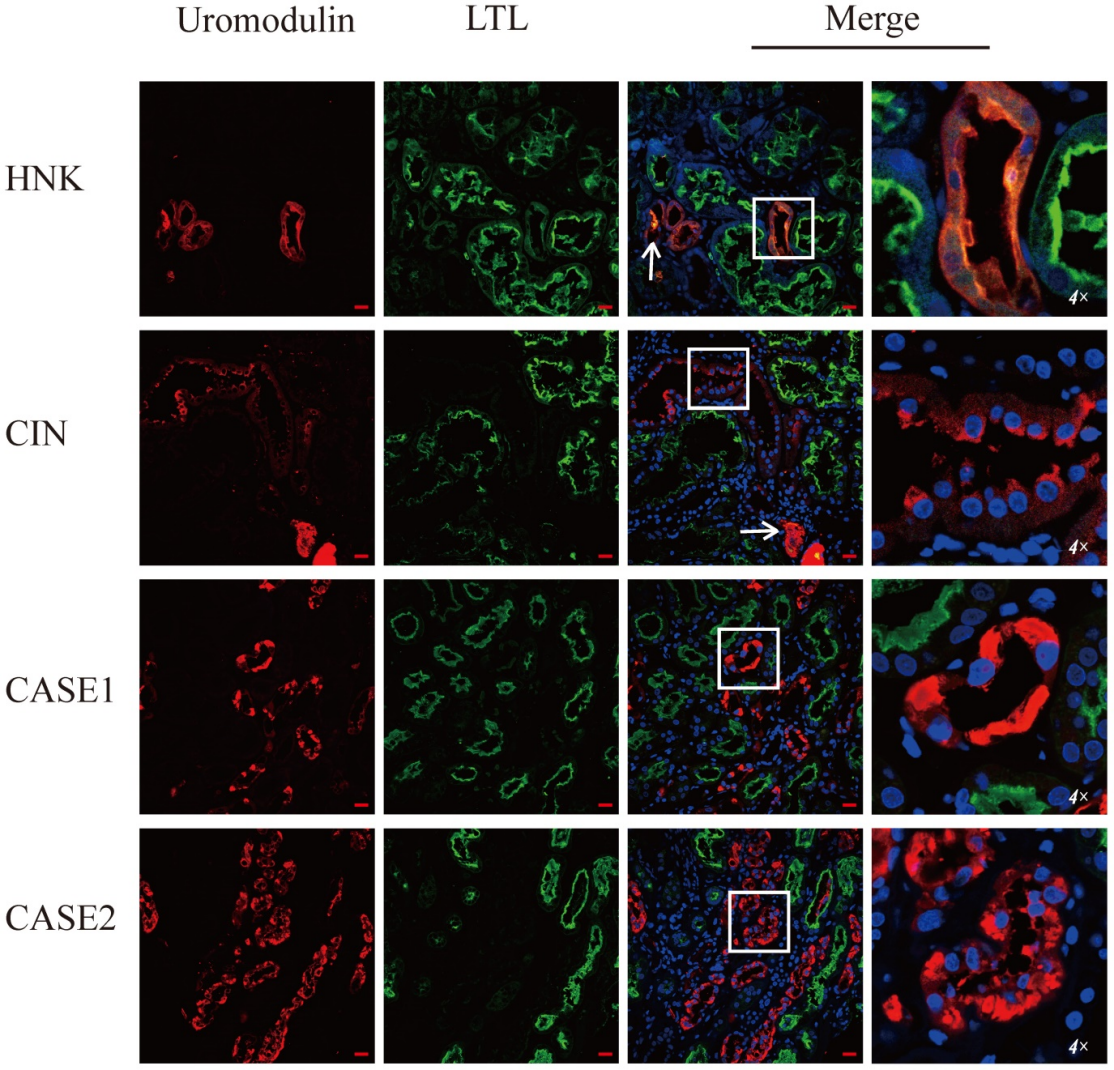
** Uromodulin expression increases in kidneys of ADTKD-UMOD cases.** Representative staining images of kidney paraffin sections from a healthy donor (HNK), sporadic chronic interstitial nephritis (CIN) case and two ADTKD-UMOD cases (CASE1, CASE2). Red, uromodulin; green, lotus tetragonolobus lectin (LTL; a marker of the brush border). Original magnification: 400×, scale bar, 20 µm; the areas in the white boxes were enlarged 4×. In HNK, uromodulin was mainly located on the cell membrane of distal tubular epithelial cells. In CIN, uromodulin was also distributed on the cell membrane, and the expression level did not significantly change compared with HNK. Secreted uromodulin protein was seen in the lumen in HNK and CIN (shown by arrows). In ADTKD-UMOD CASE1 and CASE2, there was significant uromodulin expression that aggregated in the cytoplasm of the distal tubular epithelial cells. Secreted uromodulin was not detected in ADTKD-UMOD.

Special eosinophilic “fluffy” inclusions could be seen in the epithelial cells of the thick ascending limb of Henle's loop (TALH) and the distal tubules by hematoxylin-eosin staining (HE) stained sections, which is consistent with the intracellular hyaline change under Masson-trichrome staining (Figure 1A-D). Electron microscopy images showed that there were significantly expanded rough endoplasmic reticulum (ER) and the smooth ER accompanied with the accumulation of lower electron density in them, which usually indicated the abnormal accumulation of protein (Figure 1E-G). In order to further clarify the substances in the expanded ER, specific immunofluorescent staining of uromodulin was performed in the paraffin-embedded renal sections of two probands (the renal biopsy paraffin sample of case 3 could not be obtained), one healthy donor kidney (HNK) and 23 sporadic chronic interstitial nephritis (CIN) patients. Lotus tetragonolobus lectin (LTL), a marker of the brush border of proximal tubular epithelial cells, was used to distinguish the proximal from distal tubules. The immunofluorescence staining (IF) results showed that uromodulin located specifically in the distal tubules including the TALH, evidenced by the lack of overlapping with LTL signal. No matter HNK or CIN patients' kidney samples, the expression of uromodulin in renal tubular epithelial cells were all concentrated on the apical membrane of the luminal side, with only weak expression in the cytoplasm, suggesting a polarity distribution of uromodulin. Moreover, the secreted uromodulin protein could be seen in some lumens of the HNK and CIN cases (Figure 2). In the ADTKD-UMOD cases, the expression of uromodulin increased significantly and aggregated in the cytoplasm in a clump shape without any uromodulin being detected in the lumens, suggesting that mutant uromodulin could not be transported to the cell membrane and thus accumulated in the ERs.

### ER stress induces up-regulation of CHOP in ADTKD-UMOD

To verify the ER stress induced by mutant uromodulin in ADTKD-UMOD, glucose-regulated-protein 78 (GRP78), an ER-stress marker protein, was tested. It's interesting to find though the upregulation of GRP78 could be found in all CIN patients, but only in ADTKD-UMOD patients there were colocalization of GRP78 with uromodulin. CHOP is a transcription factor downstream of the ER-stress. Unlike GRP78, the upregulation of CHOP could be found only in ADTKD-UMOD cases, not in other CIN patients, and there were also definite colocalization of CHOP with uromodulin, suggesting that the activation of CHOP is specific in the pathogenesis of ADTKD-UMOD.

### TM-induced ER stress promotes EMT and ECM deposition *in vitro*

**Figure 5:**
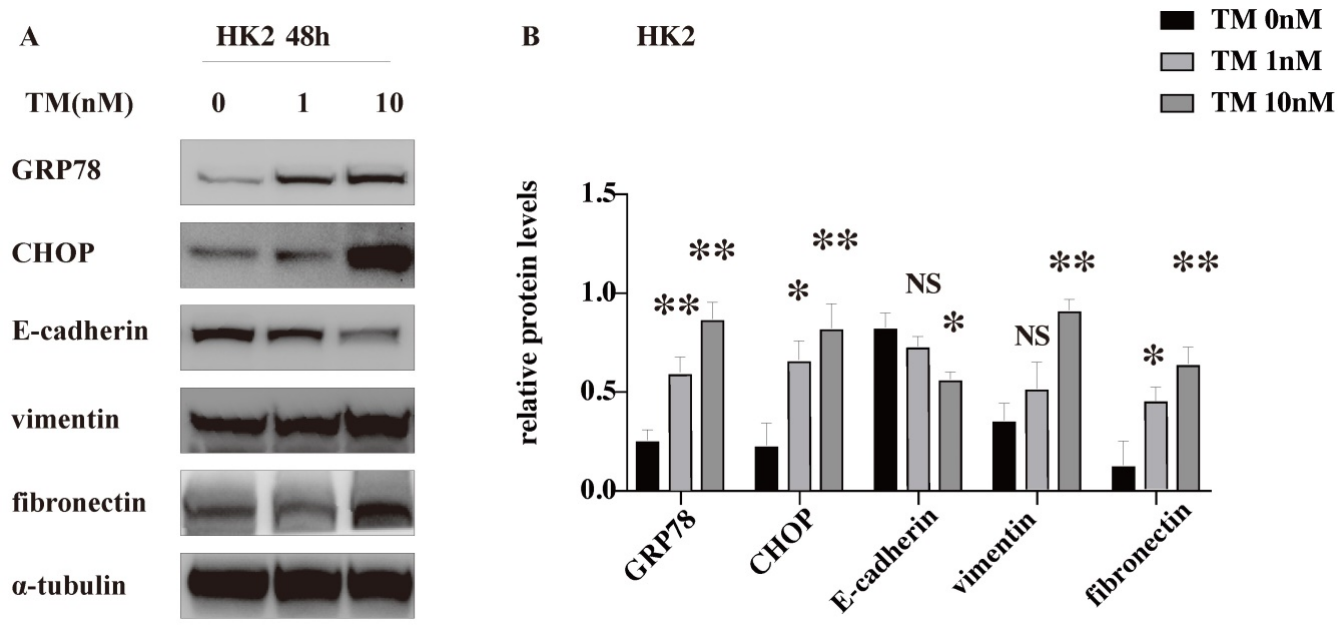
**TM-induced ER stress promotes EMT and ECM deposition *in vitro*.** ER stress was induced in HK-2 cells by tunicamycin (TM) (1 & 10 nM for 48 h). Representative western blots and quantification of the relative protein levels are presented. Experiments were repeated at least three times. Data are presented as means ± SEM. *P < 0.05, **P < 0.01, NS, not significant, all compared with the untreated group by 2-tailed Student t test.

To explore whether CHOP participates in the renal tubulointerstitial fibrosis in ADTKD-UMOD, human renal tubular epithelial (HK-2) cells were stimulated with an ER stress inducer (tunicamycin, TM). TM is an inhibitor of N-glycosylation of membrane proteins and is known to be potent inducer of ER stress signaling 21, which was used to inhibit protein glycosylation and imitate the mutant protein retention in ER to cause ER stress. The western blot results show that TM increased the expression of both GRP78 and CHOP in a dose-dependent manner, suggesting a successful activation of ER stress. The decreased protein level of E-cadherin (a marker of epithelial cell) together with the increased expression of vimentin (a marker of mesenchymal cell) suggested the occurrence of EMT. Meanwhile, fibronectin, one of the main components of extracellular matrix (ECM) was dose dependently increased with the TM-induced ER stress (Figure 5).

### CHOP knockdown restores the upregulation of vimentin and FN induced by TM

**Figure 6:**
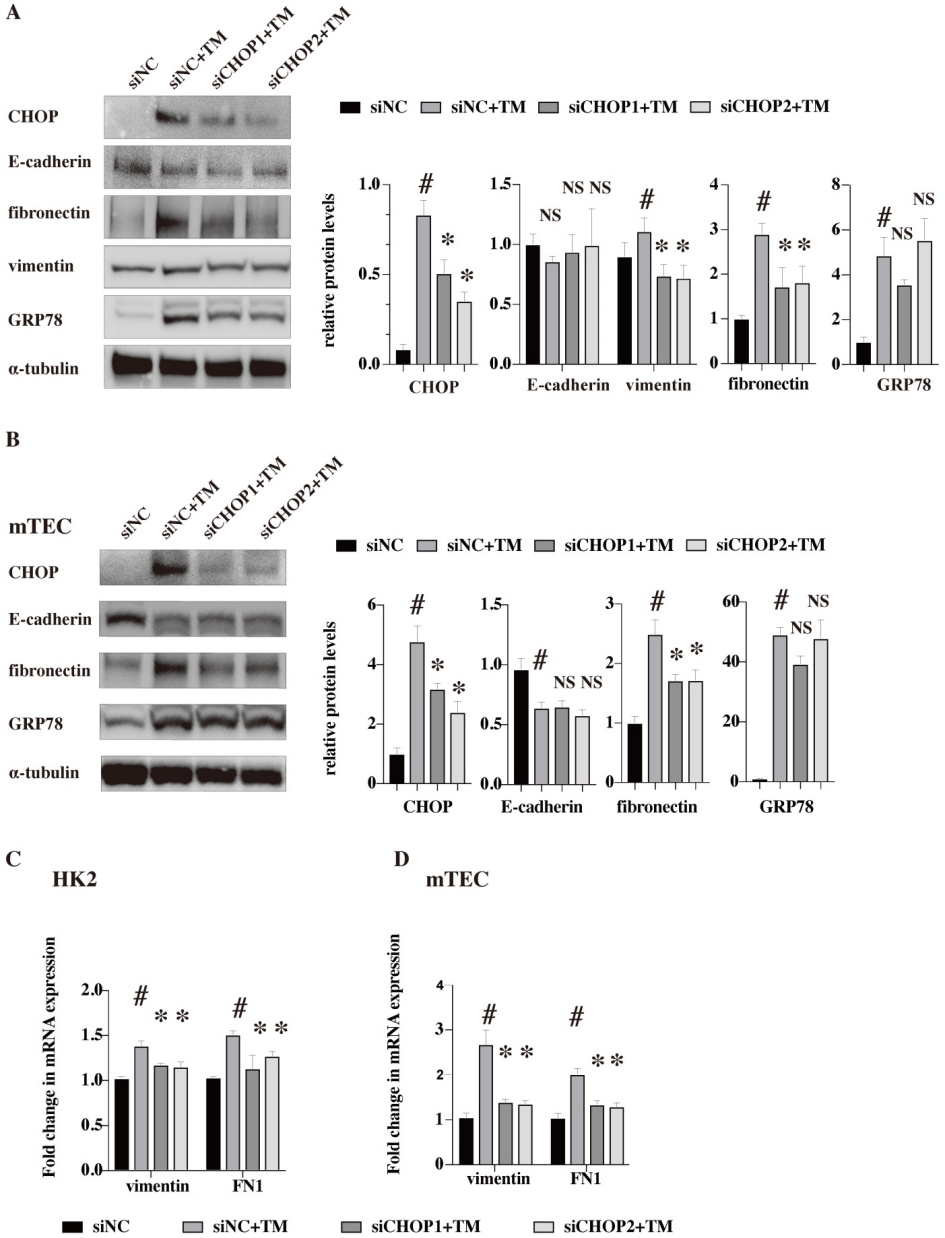
***CHOP* knockdown restores the upregulation of vimentin and FN induced by TM.** HK-2 **(A,C)** and mTEC **(B,D)** were transfected with siRNA against *CHOP* (siCHOP1 and siCHOP2) or control siRNA for 24 h, then treated with TM (HK2 in 10 nM and mTEC in 20nM for 48 h). Representative western blots and quantification of the relative protein levels are presented (A,B). Gene expression analysis of vimentin and fibronectin in the HK2 (C) and mTEC (D) were analyzed after treated with TM for 24 h. At least three independent experiments were performed.^ #^*P* < 0.05, *P < 0.05, NS, no significant.

To further clarify whether the effects of ER stress on EMT and ECM deposition were CHOP dependent, siRNA against *CHOP* (two siRNA, siCHOP1 and siCHOP2) were used to down-regulate its expression in human renal tubular epithelial cells (HK2) and mouse renal tubular epithelial cells (mTEC) (Figure 6). The western blot results showed that TM could successfully induce ER stress and upregulate the protein level of CHOP in both human and mouse renal tubular epithelial cell lines. But, *CHOP* knockdown only restored the upregulation of vimentin while had no effects on the E-cadherin expression (vimentin protein were not detected in mTEC cell lines which might be due to the cell line characters 22). Our results suggested that the EMT induced by ER stress was at least partially dependent on CHOP activation. Besides, CHOP knockdown significantly reduced the upregulation of fibronectin both in protein and mRNA levels, indicating that ECM deposition induced by ER stress is CHOP dependent. At the meanwhile, *CHOP* knockdown showed no effects on GRP78 expression (Figure 6).

## Discussion

Through UMOD genetic screening in the patients whose clinical and pathological changes were in line with ADTKD-UMOD, we found 3 cases of ADTKD-UMOD in CIN patients during the past 10 years. Among them only one case had been diagnosed correctly before and two of them were newly diagnosed with unreported missense mutations. The abnormal uromodulin accumulation in renal tubular epithelial cells could be verified by specific UMOD immunofluorescence staining. But some mild change in tubular epithelial cells deserves more attention, such as “fluffy” inclusions in HE staining and expanded ER in electron microscopy images. ER stress was verified in the uromodulin accumulated epithelial cells by the upregulation of GRP78 and CHOP which showed specifically colocalization with uromodulin. *In vitro*, TM was used to induce ER stress and the upregulation of GRP78 and CHOP could be found as well as the EMT and ECM deposition in epithelial cells. CHOP knockdown further confirmed that the EMT effects and ECM deposition induced by TM on epithelial cells are partially CHOP dependent, which is downstream of GRP78. For the first time, we showed that GRP78/CHOP activation took part in the fibrosis pathogenesis process of ADTKD-UMOD patients through EMT and ECM deposition.

In CKD caused by monogenic kidney disease, *UMOD* mutations account for 3% of the cases 23. A single-center survey in England found the incidence of ADTKD-UMOD is second to ADPKD, causing 2% of end-stage renal disease cases 24. In China, the diagnosis rate of ADTKD-UMOD is very low though there are many young people with hyperuricemia. Only a few reports about ADTKD-UMOD had been retrieved, and 13 different *UMOD* pathogenic mutations were found in 15 Chinese families 4, 17, 25, 26. Since China had implemented a family planning policy, the phenomenon of many only-children makes it difficult to obtain family history of genetic diseases. More attention should be paid on the pathological abnormalities in tubules, such as eosinophilic “fluffy” inclusions or expanded endoplasmic reticulum. These finding combined with other classic changes may remind us of ADTKD-UMOD in time. Of course, genetic screening is still needed for these patients to confirm the diagnosis which is the gold standard of the disease 1.

UMOD mutation sites reported previously are concentrated in exons 3 and 4, which encode three epidermal growth factor-like regions and one cystine-rich region and are related to protein folding and transport. Additionally, cysteine residues in the amino acid sequence of uromodulin are highly conserved 27, playing an important role in the formation of intramolecular disulfide bonds 2. Mutations at these sites reduce the protein's transport ability in the cytoplasm. The two novel missense mutations both located in exon 3 and caused the substitution of conserved cysteine residues (C315S, C126Y), which affected the formation of intramolecular disulfide bonds and in turn the uromodulin protein was accumulated in ER 28. The substitution of cysteine by tyrosine at C315 (C315Y) has also been reported in ADTKD-UMOD 29. Another mutation T605G is located in exon 9 within the glycosylphosphatidylinositol (GPI) anchor signal segment of uromodulin, thus it may cause the accumulation of uromodulin by affecting the function of the GPI anchor 17.

Uromodulin, coded by the *UMOD* gene, also known as Tamm-Horsfall protein, is the most common protein in the urine of healthy individuals and is mainly secreted by epithelial cells of the TALH with a low expression level in the distal convoluted tubules 30, 31. The main function of uromodulin is related to the regulation of calcium oxalate excretion and the function of the Na^+^/K^+^/Cl^-^ cotransporter, NKCC2. It has been reported that the abnormal accumulation of uromodulin in renal tubules verified by immunohistochemistry or fluorescence may suggest the diagnosis of ADTKD-UMOD 32-34. But as we tested the expression of uromodulin in all other 20 CIN patients, we found the high expression of uromodulin is not specific, but the loss of polarity in apical cell membrane, as well as the lack of secreted uromodulin in the tubular lumen may be clues for further genetic screening.

At present, it is generally believed that ER stress is an important pathogenic mechanism of ADTKD-UMOD. GRP78, also referred to as BiP, is an ER chaperone playing an essential role in unfolded protein response (UPR) and often used as an ER stress marker. Consistent with our results, the upregulated expression of GRP78 had been found in ADTKD-UMOD kidney tissues 32. Our results not only confirmed their finding, but also showed a strong colocalization of GRP78 with UMOD. Considering the function of GRP78, both prior researches and our results suggested that under ER stress, GRP78 may detach from the ER stress sensory receptor molecules on the ER membrane, then bind to unfolded or misfolded UMOD proteins to activate the UPR and promote the restoration of proteostasis, which is an adaptive response to cell stimulators. However, under excessive and prolonged ER stress caused by the misfolded uromodulin, proteostasis cannot be restored and GRP78 continues to be highly expressed 35. There are also other stimulators that could cause ER stress and the upregulation of GRP78, like sporadic chronic interstitial nephritis, but under this condition GRP78 would not be colocalized with UMOD.

GRP78 affects tumor metastasis and development through EMT in many tumors. Studies have found that overexpression of GRP78 can promote the up-regulation of fibronectin, down-regulation of E-cadherin, and promote the expression and secretion of TGF-β1, thereby affecting EMT 36. In our study, CHOP knockdown suppresses EMT, but has no effect on the expression of GRP78, suggesting that CHOP mediates EMT which should be downstream of GRP78 activation.

**Figure 7:**
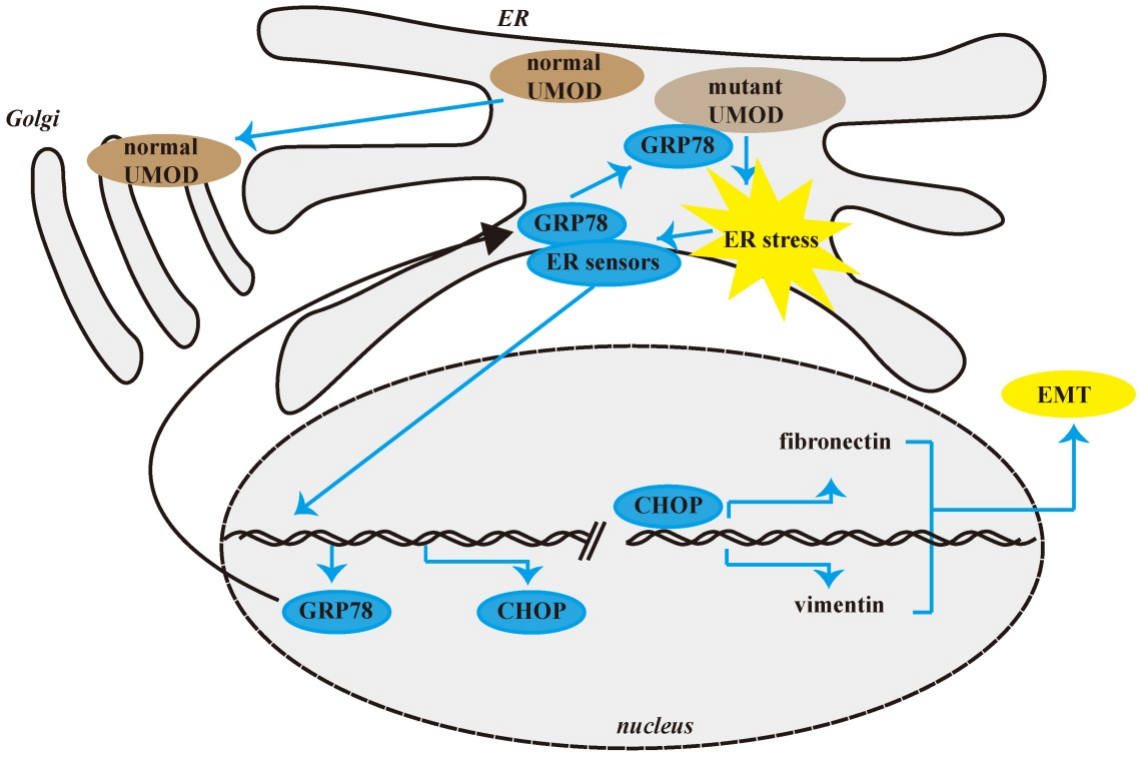
** Schematic diagram of CHOP promoting renal interstitial fibrosis in ADTKD-UMOD.** Under normal physiological conditions, UMOD is synthesized and transported to the Golgi apparatus for processing, and then transported to the cell membrane. However, in ADTKD-UMOD, when mutant UMOD accumulates in the ER, it causes ER stress, which activates the ER membrane sensors. Consequently, GRP78 separates from the sensors and binds to the mutant UMOD to promote its folding; simultaneously the stress signal is transferred to the nucleus, resulting in high expression of CHOP and other ER chaperones (mainly GRP78). As a transcription regulator, CHOP promotes the expression of fibronectin and vimentin, which in turn cause the formation of renal interstitial fibrosis.

As a transcription factor, CHOP plays an important role in endoplasmic reticulum stress-mediated apoptosis 37-39. Under physiological conditions, CHOP is ubiquitously expressed at very low levels. Our study found that the expression of CHOP was specifically upregulated in the renal tubular epithelial cells of ADTKD-UMOD patients but not in any sporadic chronic interstitial nephritis patients. The colocalization of upregulated CHOP with accumulated UMOD in the same tubular epithelial cells suggests the activation of CHOP is involved in the pathogenesis of UMOD. Consistent with our results, an earlier study showed that the expression of CHOP was increased in the kidneys of mice with a *UMOD* point mutation 2. Through *in vitro* experiments, we further confirmed that the ER stress could induce EMT of the tubular epithelial cells, and this effect is partially CHOP dependent (Figures 6 & 7). It has been reported that CHOP could promote the transcription of growth differentiation factor 15 (GDF15), thereby promoting the occurrence of EMT in Human colorectal cancer cell lines 40. But this is the first time to show that CHOP participates in the EMT of tubular epithelial cells in renal fibrosis.

Although our research was based on the clinical findings and then verified the relevant mechanisms *in vitro*, there are still some shortcomings. First, though the low incidence rate, more cases are still needed to verify our findings; Second, more definite and direct proof are still needed, like using tubular cells with *UMOD* point mutations to prove that the mutations cause ER stress and then EMT *in vitro*; and third, more specific molecular mechanism need to be explored, like whether CHOP influences EMT-related transcription factors, such as Snail and Twist1 41.

Through genetic screening of people suspected of ADTKD, two new mutation sites of UMOD were discovered and two patients with mutations at that site were newly diagnosed. Through the analysis of kidney pathological changes and UMOD-specific staining in confirmed patients, we found and confirmed that CHOP activation participates in the mechanism of uromodulin accumulation-induced renal fibrosis by promoting the EMT process of tubular epithelial cells and ECM deposition.

## Supplementary Material

Supplementary files.

## Figures and Tables

**Figure 3:**
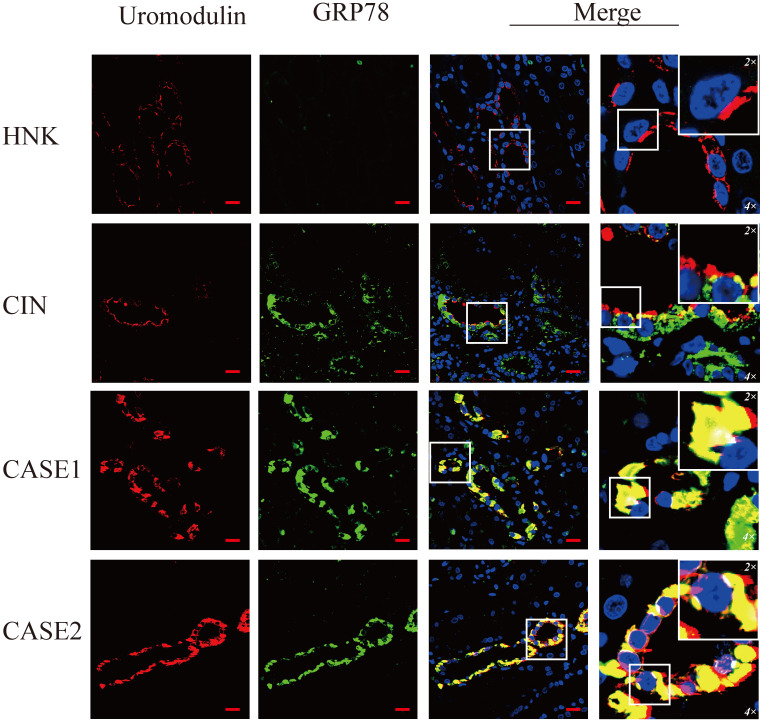
** GRP78 is highly expressed and colocalizes with uromodulin in ADTKD-UMOD cases.** Representative staining images of kidney sections from HNK, CIN, CASE1 and CASE2 [uromodulin (red), GRP78 (green) and DAPI (blue)]. GRP78 was expressed at low levels in tubular epithelial cells of HNK, while it showed an obvious increase in CIN, but it rarely colocalized with uromodulin. In ADTKD-UMOD, the high expression of GRP78 was distinctive in UMOD-producing tubular cells and colocalized with uromodulin. Original magnification: 400×; scale bar, 20 µm; areas in white boxes are magnified 4× and then 2× from the original images.

**Figure 4:**
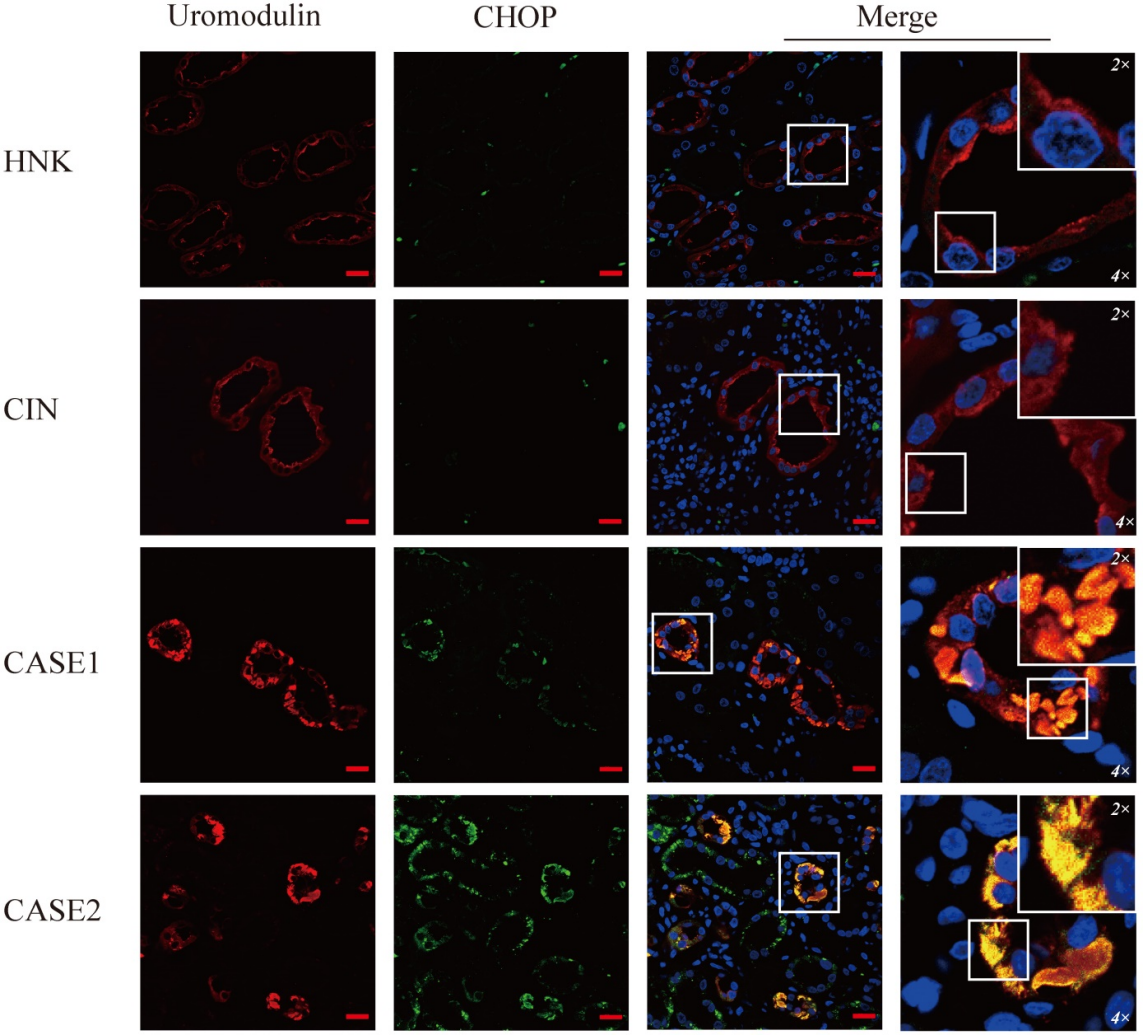
** CHOP is specifically expressed in UMOD-producing tubular cells of ADTKD-UMOD cases.** There were a few punctate fluorescent dots of CHOP (green) localized at individual tubular cells of HNK and CIN, compared with distinctive increased expression of CHOP and obvious colocalization with UMOD (red) in both ADTKD-UMOD cases. Original magnification: 400×; scale bar, 20 µm; areas in white boxes are enlarged 4× and then 2× from the original images.

**Table 1:** *UMOD* mutations in patients

ID	Mutation	EXON	Nucleotide change	Variant of coding sequence	PROVEAN^#^ score	Prediction (cutoff= -2.5*)
CASE1	1	3	c.944G>C	Cysteine>serine (C315S)	-8.569	Deleterious
CASE2	2	3	c.377G>A	Cysteine>tyrosine (C126Y)	-5.146	Deleterious
CASE3	3	9	c.1815A>G	Threonine>Glycine (T605G)	-3.714	Deleterious

# It refers to “Protein Variation Effect Analyzer (PROVEAN)” used to predict the functional effects of protein sequence variations. *PROVEAN score -2.5 is the default score threshold, with sensitivity and specificity of 79.76 and 78.63 respectively for prediction 16.

**Table 2:** Clinical features of patients with *UMOD* mutations

ID	CASE 1	CASE 2	CASE 3
Gender	male	male	female
Age at diagnosis	22	31	39
Family history of CKD	NO	Yes	Yes
Serum uric acid (μmol/L)	369	385	415
Onset age of hyperuricemia	22	21	29
Gout	NO	Yes	NO
Fractional excretion of uric acid (%)	-	1.87	5.42
Creatinine (μmol/L)	126	156	149
EGFR (ml/min per 1.73 m^2^)	66.0	48.1	35.92
CKD stage (1-5)	2	3	3
Proteinuria	negative	negative	negative
Hematuria	negative	negative	negative
24h proteinuria (g)	0.125	0.065	-
Renal ultrasound (length×width (thickness))	left: 10.2×4.7(1.8) right: 10.3×2.6(1.0)	left: 9.2×5.3(1.4) right: 9.1×3.6(1.1)	normal size
renal biopsy			
glomerulosclerosis	3/18	10/21	16/22
renal tubular epithelial cells	vacuolar and granular degeneration	vacuolar and granular degeneration;	vacuolar and granular degeneration
Tubule atrophy	Multiple small focal	Multiple focal (15%)	Multiple small focal
renal interstitium	multifocal fibrosis	multifocal fibrosis	multifocal fibrosis
inflammatory cell infiltration	not obvious	A small amount of mononuclear cells	A small amount of mononuclear cells and individual eosinophils
immunofluorescence	all negative	IgM±,C3+,C1q±, with diffuse segmental distribution and granular deposition in the mesangial area; IgG, IgA and Fg are negative	all negative
uric acid crystal deposits	not obvious	not obvious	not obvious
diagnosis of biopsy	interstitial fibrosis	interstitial fibrosis	interstitial fibrosis
electron microscope	-	Bundled and cystic endoplasmic reticulum by EM	-

“-” refers to no examination.

## Abbreviations

CHOP: C/EBP-Homologous Protein
EMT: epithelial to myofibroblast transformation
ADTKD: autosomal dominant tubulointerstitial kidney disease
UMOD: uromodulin
ESRD: end-stage renal disease
ER: endoplasmic reticulum
TALH: thick ascending limb of Henle's loop
PROVEAN: Protein Variation Effect Analyzer
HNK: healthy donor kidney
CIN: sporadic chronic interstitial nephritis
LTL: lotus tetragonolobus lectin
UPR: unfolded protein response
TM: Tunicamycin
siRNA: small interference RNA
HK2: human renal tubular epithelial cells
mTEC: mouse renal tubular epithelial cells
