# Supplementary material for: Upregulation of C/EBP Homologous Protein induced by ER Stress Mediates Epithelial to Myofibroblast Transformation in ADTKD-UMOD
Source: Int J Med Sci. 2022 Jan 24;19(2):364–76. doi: 10.7150/ijms.65036 (PMC8795802; doi:10.7150/ijms.65036)
Supplement: Supplementary file 1 — Supplementary files. [file ijmsv19p0364s1.pdf]

Supplement File 1: Information of the three cases

In patient 1, a 22-year-old man, we detected the first novel missense mutation (c.944G>C). By reviewing the medical history of the proband, we found that his mother and two uncles had a history of hyperuricemia and gout for many years, and one of the uncles passed away due to unknown reasons， while the creatinine levels of the others remained around 100 μmol/L. We further performed a *UMOD* exon test on his mother and found that she had the same mutation. Physical examination of the proband revealed elevated blood uric acid (600 μmol/L) and serum creatinine (170 μmol/L), without proteinuria or hematuria, and no other abnormalities in the urine examination. Renal ultrasound showed that both kidneys were normal in size and left renal vein compression was seen. Pathology of the kidney biopsy showed interstitial nephritis. After treatment of the hyperuricemia, the serum uric acid returned to normal (369 μmol/L) and the serum creatinine was around 120 μmol/L.

In patient 2, a 31-year-old man, the mutation, c.377G>A, was detected, resulting in the amino acid change, p.C126Y (amino acid 126 was changed from cysteine to tyrosine). The patient's mother, uncle and grandmother had a history of hyperuricemia and chronic renal failure. The uncle and grandmother died of uremia. The DNA of the relatives available was tested at the same locus, and the same mutation was found in the proband's mother, son and cousin. However, no mutation at this position was detected in his father. The proband had a history of hyperuricemia in the past 10 years. At the time of kidney puncture, his serum creatinine was 156 μmol/L. Both kidneys were normal in size as assessed by ultrasound examination, and there was no proteinuria or hematuria. Renal pathology showed interstitial nephritis and hyperuricemic nephropathy was diagnosed by a clinician.

In patient 3, a 39-year-old female, we detected the missense mutation: c.1815 A>G, p.T605G (threonine changed by glycine). Her brother has been accurately diagnosed in our hospital by genetic testing. Genetic testing of seven relatives including this case revealed the same pathogenic mutation. At the age of 29, the patient was diagnosed with hyperuricemia, with blood uric acid of 700 μmol/L and a normal level of serum creatinine. Physical examination at 36 years of age found that the blood creatinine was 119 μmol/L. At the time of admission to the hospital, her blood uric acid was 415 μmol/L. Her kidneys were of normal size, without proteinuria and hematuria, and the pathological diagnosis was chronic interstitial nephritis.

Supplement File 2:

Mouse Primer Name	sequence(5'to3')
mβ-actin-F	CATTGCTGACAGGATGCAGAAGG
mβ-actin-R	TGCTGGAAGGTGGACAGTGAGG
mchop-F1	CTGGAAGCCTGGTATGAGGAT
mchop-R1	CAGGGTCAAGAGTAGTGAAGGT
mFN-F	ATGTGGACCCCTCCTGATAGT
mFN-R	GCCCAGTGATTTCAGCAAAGG
mGRP78-F	TGTCTTCTCAGCATCAAGCAAGG
mGRP78-R	CCAACACTTCCTGGACAGGCTT
mN-cadherin-F	CCTCCAGAGTTTACTGCCATGAC
mN-cadherin-R	CCACCACTGATTCTGTATGCCG
mE-cadherin-F	GGTCATCAGTGTGCTCACCTCT
mE-cadherin-R	GCTGTTGTGCTCAAGCCTTCAC
mvimentin-F	CGGAAAGTGGAATCCTTGCAGG
mvimentin-R	AGCAGTGAGGTCAGGCTTGGAA

Human Primer Name	sequence(5'to3')
hβ-actin-F	CACCATTGGCAATGAGCGGTTC
hβ-actin-R	AGGTCTTTGCGGATGTCCACGT
hFN-F	CGGTGGCTGTCA GTCAAAG
hFN-R	AAACCTCGGCTTCCTCCATAA
hCHOP-F	GGAAACAGAGTGGTCATTCCC
hCHOP-R	CTGCTTGAGCCGTTCATTCTC
hGRP78-F	CATCACGCCGTCCTATGTCG
hGRP78-R	CGTCAAAGACCGTGTTCTCG
hN-cadherin-F	CCTCCAGAGTTTACTGCCATGAC
hN-cadherin-R	GTAGGATCTCCGCCACTGATTC
hE-cadherin-F	GCCTCCTGAAAAGAGAGTGGAAG
hE-cadherin-R	TGGCAGTGTCTCTCCAAATCCG
hvimentin-F	AGGCAAAGCAGGAGTCCACTGA
hvimentin-R	ATCTGGCGTTCCAGGGACTCAT
